# Dehydroabietic Acid Derivative QC4 Induces Gastric Cancer Cell Death via Oncosis and Apoptosis

**DOI:** 10.1155/2016/2581061

**Published:** 2016-02-29

**Authors:** Dongjun Luo, Qing Ni, Anlai Ji, Wen Gu, Junhua Wu, Chunping Jiang

**Affiliations:** ^1^Department of Hepatobiliary Surgery, Nanjing Drum Tower Hospital Clinical College of Nanjing Medical University, Nanjing, Jiangsu 210008, China; ^2^Department of General Surgery, The Second Clinical Medical School of Yangzhou University, Jiangsu 225000, China; ^3^Department of General Surgery, Frist People's Hospital of Yangzhou, Yangzhou, Jiangsu 225000, China; ^4^College of Chemical Engineering, Nanjing Forestry University, Nanjing, Jiangsu 210037, China; ^5^Jiangsu Key Laboratory of Molecular Medicine, Medical School, Nanjing University, Nanjing, Jiangsu 210093, China; ^6^Department of Hepatobiliary Surgery, Affiliated Drum Tower Hospital of Nanjing University Medical School, Nanjing, Jiangsu 210008, China

## Abstract

*Aim*. QC4 is the derivative of rosin's main components dehydroabietic acid (DHA). We investigated the cytotoxic effect of QC4 on gastric cancer cells and revealed the mechanisms beneath the induction of cell death.* Methods*. The cytotoxic effect of QC4 on gastric cancer cells was evaluated by CCK-8 assay and flow cytometry. The underlying mechanisms were tested by administration of cell death related inhibitors and detection of apoptotic and oncosis related proteins. Cytomembrane integrity and organelles damage were confirmed by lactate dehydrogenase (LDH) leakage assay, mitochondrial function test, and cytosolic free Ca^2+^ concentration detection.* Results*. QC4 inhibited cell proliferation dose- and time-dependently and destroyed cell membrane integrity, activated calpain-1 autolysis, and induced apoptotic protein cleavage in gastric cancer cells. The detection of decreased ATP and mitochondrial membrane potential, ROS accumulation, and cytosolic free Ca^2+^ elevation confirmed organelles damage in QC4-treated gastric cancer cells.* Conclusions*. DHA derivative QC4 induced the damage of cytomembrane and organelles which finally lead to oncosis and apoptosis in gastric cancer cells. Therefore, as a derivative of plant derived small molecule DHA, QC4 might become a promising agent in gastric cancer therapy.

## 1. Introduction

Gastric cancer is a frequent malignant tumor in digestive system, although its incidence has declined over the past several years in developed countries on account of improvement of people's lifestyle and environment; it remains a major public health burden as the fifth most common cancer and the third material cause of cancer death worldwide especially in Eastern Asia (i.e., China) [1]. So far, surgery remains the most curative therapy for gastric cancer. Nevertheless, for symptoms of early gastric cancer are frequently less or atypical, a substantial proportion of patients present at advanced stage and lost the opportunity of radical gastrectomy when they see doctors. Unfortunately again, local recurrence or distant metastasis occurs in over half of patients after radical resection. Treatments for advanced and recurrent gastric cancer incorporate chemotherapy, radiotherapy, and biological therapy. Whereas their outcomes are always unsatisfactory for drug resistance and side effects. Up to now, the 5-year survival of metastatic gastric cancer patients is not exceeding 10% [2, 3]. Hence, it is urgent to develop new cytostatic agents and valid treatments for gastric cancer.

Natural compounds from plants and their derivatives should be a vital resource for developing new anticancer drugs. Interests in natural products research are strong and can be attributed to several causes: unmet therapeutic needs, the diversity of chemical structures and biological activities, and the development of novel techniques to detect biologically active natural products [4]. Treatments of many malignancies by natural compounds both in the form of their natural forms or acting as a template for synthetic modifications have been used extensively in clinic [5]. As we all know, taxol and vincristine which have been widely used to treat breast, ovary, lung cancer, and so forth clinically are, respectively, abstracted from taxus and* Catharanthus roseus*. Traditional Chinese medicine (TCM) is an empirical medicine that uses herbal medicine as the main treatment. Along with the advancement in pathology of neoplastic disease and oncotherapy, TCM can become the resource for novel drug discovery and development. And it is a convenient and efficient method of discovering and developing new anticancer drugs from Chinese herbal medicine and its derivatives.

Rosin is produced by removing turpentine from oleoresin of* Pinus* species and it is a commonly used medicine in TCM [6]. Besides its applications in chemical industries, the biological activities have been revealed in recent decades [7, 8]. DHA is one of the main resin acids and it is a promising template for synthetic modifications. DHA derivatives have been reported to have antibacterial and antiproliferative activities. Gu et al. synthesized and evaluated the antibacterial effect of some novel N-acylhydrazone derivatives of DHA [9]. Walter Pertino et al. synthesized several triazole derivatives from DHA and found antiproliferative effect on many tumor cell lines including gastric epithelial adenocarcinoma cell lines [10]. Huang et al. synthetized a series of dipeptide derivatives from DHA and screened the antitumor activities of these compounds. They found that many DHA derivatives showed moderate-to-robust inhibitory effects against tumor cells [11]. The work by Zhang et al. studied the cytotoxic effect of N-substituted 1H-dibenzo [a, c]carbazole derivative of DHA on hepatocellular carcinoma (HCC) cells and concluded that this derivative could significantly inhibit HCC cell viability [12]. These research achievements proved the potential biological activities of DHA derivatives. Nevertheless, it is worth further studying their anticancer effect.

In the present study, we studied the biological activity of a novel DHA derivative QC4 and investigated whether QC4 exerted antitumor effects against gastric cancer cells. Furthermore, we focused on the specific cell death type caused by QC4 in gastric cancer cells.

## 2. Materials and Methods

### 2.1. Synthesis of DHA Derivative QC4

**Figure 1:**
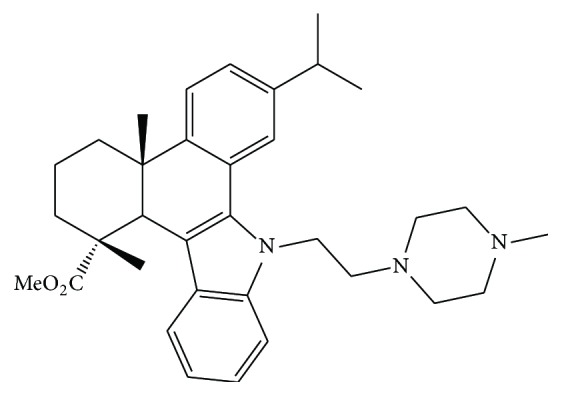
Chemical structure of QC4.

QC4 is an N-substituted 1H-dibenzo [a,c]carbazole derivative synthesized from dehydroabietic acid. The chemical structure of QC4 is illustrated in Figure 1 and the synthetic route is detailed in the paper of Gu and coworkers [9].

### 2.2. Cells and Reagents

The human gastric cancer cell lines SGC-7901 and MGC80-3 were purchased from Cell Bank of Chinese Academy of Sciences (Shanghai, China). All the cells were maintained in Dulbecco's Modified Eagle Medium (DMEM, WISENT, CA) containing 10% fetal bovine serum (FBS) (GIBCO BRL, Gaithersburg, MD) in a humidified atmosphere at 37°C and 5% CO_2_. Antibodies against calpain-1, caspase-3, cleaved caspase-3, caspase-9, cleaved caspase-9, poly(ADP-ribose) polymerase (PARP), and cleaved PARP were purchased from Cell Signaling Technology (Beverly, MA). Antibodies against *α*-tubulin, *β*-actin, and glyceraldehyde-3-phosphate dehydrogenase (GAPDH) were obtained from Bioworld Technology Inc. (Bioworld, USA).

### 2.3. Cell Proliferation Assay

Cells were plated on 96-well plates at the density of 5 × 10^3^ cells per well and allowed to attach overnight. The cells were treated with QC4 for indicated time. Thereafter, CCK-8 (Dojindo Laboratories, Japan) was added to each well and incubated for 2 h. Absorbance values at 490 nm were recorded using the microplate reader. All experiments were performed in triplicates. The cytotoxicity of QC4 to each cell line was determined by the preceding results.

### 2.4. Annexin V-FITC/Propidium Iodide (PI) Double Labeling Method

After administration of QC4 for indicated time, cells were harvested and collected by centrifugation. Cells were washed with phosphate buffering solution (PBS) and resuspended in 500 *μ*L binding buffer. Then, cells were added with 5 *μ*L Annexin V-FITC and 5 *μ*L PI (Beyotime, Nantong, China) in the dark for 10 min and were subjected to flow cytometry analysis.

### 2.5. Western Blotting

To determine the level of indicated proteins, cell lysates prepared after administration of QC4 were immunoblotted as published protocol [13]. The signal was developed with ECL (Millipore, Switzerland) after incubation with appropriated second antibody.

### 2.6. Immunocytochemistry

Cells were seeded on cover glass in 24-well plate and cultured in the presence of QC4 for indicated time. They were washed with cold PBS and fixed with 4% paraformaldehyde at room temperature for 30 min. After blocking with blocking buffer (1 × PBS/5% normal serum/0.3% Triton*™* X-100) for 60 min, primary antibody was added to the wells and incubated overnight at 4°C. The specimens were incubated in fluorochrome-conjugated secondary antibody for 2 hours at room temperature in the dark. DAPI or PI staining was carried out for the manifestation of the cell nucleus. Cells on cover glass were subjected to fluorescence microscopic examination.

### 2.7. Lactate Dehydrogenase (LDH) Leakage Assay

The cell membrane integrity was determined by LDH leakage assay by using a LDH assay kit (Beyotime, Nantong, China). In brief, cells were plated on 96-well plates at the density of 5 × 10^3^ cells per well and allowed to attach overnight. After being incubated with QC4 for indicated time, the supernatants were collected and centrifuged at the speed of 1,000 rpm and were subjected to LDH detection. The absorbance at the length of 490 nm was measured by a microplate reader.

### 2.8. Transmission Electron Microscopy

The ultramicrostructural analysis of QC4-treated cells was conducted following published protocols [12]. The ultrathin sections were obtained, stained with 1% toluidine blue, and observed by a transmission electron microscope (JEM-1010, Japan).

### 2.9. Intracellular ATP Detection

Cells were cultured in the presence of QC4 for indicated time and were used to measure the concentration of intracellular ATP with a commercial ATP detection kit (Beyotime, Nantong, China) following the manufacturer's instructions. Intracellular ATP levels were taken as the luciferase activity by Dual-Luciferase Reporter Assay System.

### 2.10. Mitochondrial Membrane Potential Detection

The mitochondrial membrane potential was measured by mitochondrial membrane potential detection kit (Beyotime, Nantong, China) according to the manufacturer's protocols. Δ*ψ*m was manifested by the fluorescence intensity using the Cell Quest software.

### 2.11. Reactive Oxygen Species (ROS) Assay

The ROS level was measured by Reactive Oxygen Species Assay Kit (Beyotime, Nantong, China) according to the manufacturer's protocols. The ROS level was manifested by the fluorescence intensity of DCF.

### 2.12. Cytosolic Free Ca^2+^ Concentration Detection

The cytosolic free Ca^2+^ concentration was measured by Fluo-3 AM kit (Beyotime, Nantong, China) according to the manufacturer's protocols. The Ca^2+^ concentration was calculated through the fluorescence intensity detected by flow cytometry.

### 2.13. Statistical Analysis

All the data were expressed as mean ± SD from three individual experiments. Differences between groups were determined by using Student's *t*-test and one-way ANOVA. *p* < 0.05 was considered statistically significant.

## 3. Results

### 3.1. QC4 Exerted Cytotoxicity against Gastric Cancer Cells

**Figure 2:**
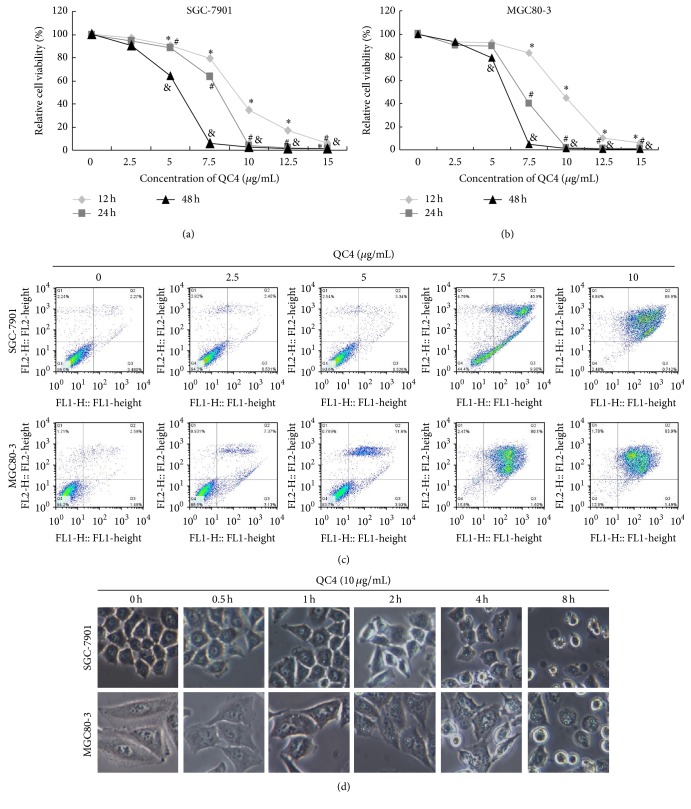
QC4 induced cell death in gastric cancer cells. (a) SGC-7901 cells were incubated with QC4 of indicated concentrations for indicated time, QC4 impaired cell viability time- and dose-dependently. (b) MGC80-3 cells were incubated with QC4 of indicated concentrations for indicated time, QC4 impaired cell viability time- and dose-dependently. (c) Flow cytometric analysis of SGC-7901 and MGC80-3 after QC4 administration at indicated concentrations for 24 h. (d) Morphological change of SGC-7901 and MGC80-3 after QC4 administration at 10 *μ*g/mL for indicated time (^*∗*^ *p* < 0.05 for QC4 treatment compared with the DMSO treated control group for 12 h; ^#^ *p* < 0.05 for QC4 treatment compared with the DMSO treated control group for 24 h; ^&^ *p* < 0.05 for QC4 treatment compared with the DMSO treated control group for 48 h).

The cytotoxic effect of QC4 on gastric cancer cell line SGC-7901 and MGC80-3 was evaluated by CCK-8 assay and flow cytometry. The inhibitory effect was also evaluated by using normal gastric epithelial cell line GES-1. We found that QC4 inhibited cell proliferation dose- and time-dependently in both SGC-7901 and MGC80-3 cells (Figures 2(a) and 2(b)). The IC_50_ values of QC4 on two cell lines for different incubation time were summarized in Table 1. The similar results were obtained by using flow cytometry method and QC4 at the concentration of over and equal to 7.5 *μ*g/mL induced severe cell death (Figure 2(c)). During the cytotoxicity determination, the morphological changes of gastric cancer cells after QC4 administration were obtained by microscopy at indicated time. As illustrated in Figure 2(d), QC4 impaired cell membrane and caused cell swelling after plasma membrane blebbing appeared in both SGC-7901 and MGC80-3 cells.

### 3.2. QC4 Destroyed Gastric Cancer Cell Membrane Integrity

**Figure 3:**
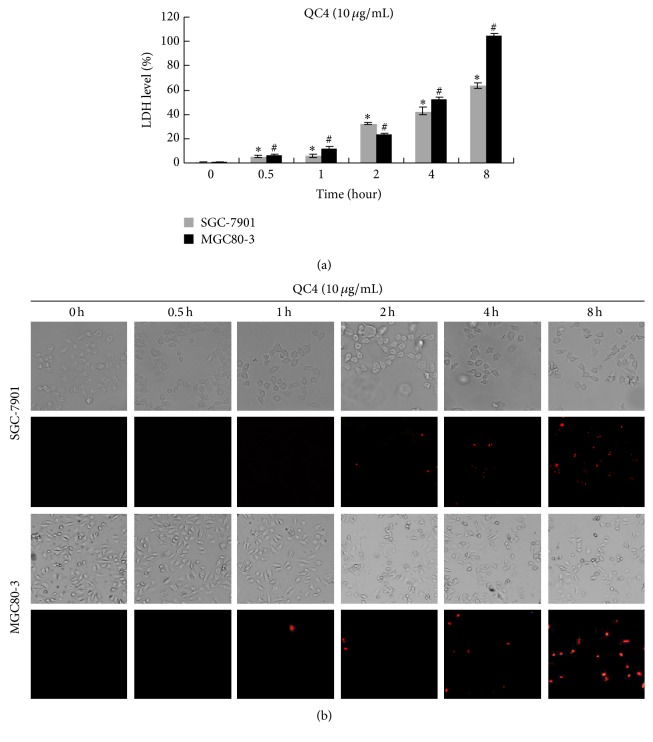
QC4 destroyed gastric cancer cell membrane integrity. (a) SGC-7901 and MGC80-3 were incubated with QC4 for indicated time at 10 *μ*g/mL, and the culture supernatants were subjected to LDH leakage analysis. (b) PI uptake assay was performed after QC4 administration. QC4 increased PI uptake in SGC-7901 and MGC80-3 cells after QC4 administration for indicated time at 10 *μ*g/mL (^*∗*^ *p* < 0.05 for QC4 treatment compared with the DMSO treated control group in SGC-7901; ^#^ *p* < 0.05 for QC4 treatment compared with the DMSO treated control group in MGC80-3).

LDH would be released into the medium and the nucleus would become easily stained by PI when the cell membrane is destructed. Hence, the destroyed cell membrane was confirmed by LDH leakage assay and PI staining. SGC-7901 and MGC80-3 were incubated with QC4 for indicated time. It turned out that QC4 increased LDH level in culture supernatants after 0.5 h, and the LDH level continued to increase to almost 60 times for SGC-7901 and 100 times for MGC80-3, respectively, after 8 h QC4 treatment which suggested a membrane rupture (Figure 3(a)). The time-dependent PI uptake was in accordance with the LDH release. As shown in Figure 3(b), both SGC-7901 and MGC80-3 cells became swelled and red fluorescence stained by PI after 1 h while no fluorescence was seen before 1 h in QC4-treated cells at 10 *μ*g/mL.

### 3.3. QC4 Induced Oncosis and Apoptosis in Gastric Cancer Cells

**Figure 4:**
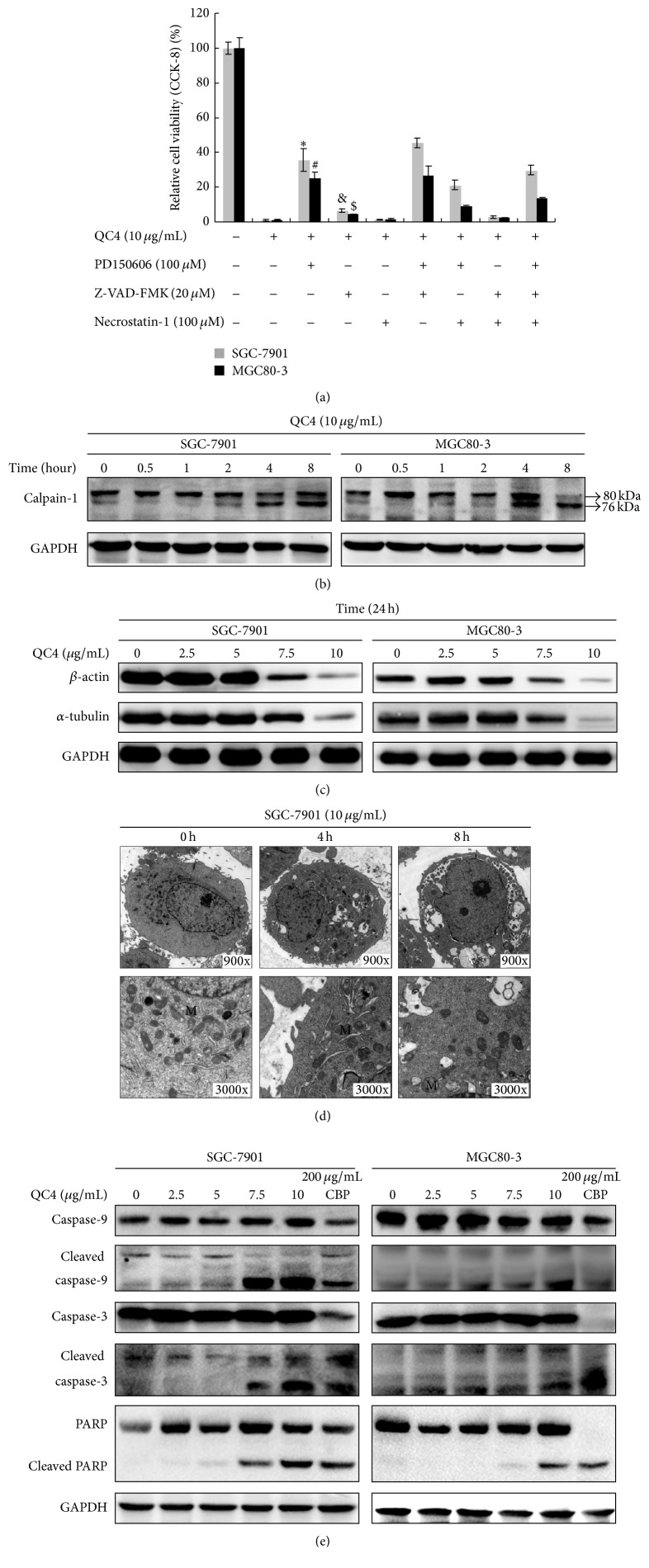
QC4 induced oncosis and apoptosis in gastric cancer cells. (a) PD150606 and Z-VAD-FMK partially reversed QC4 induced cell death. SGC-7901 and MGC80-3 cells were incubated with PD150606, Z-VAD-FMK, or Necrostatin-1 at indicated concentrations for 24 h alone or in combination with QC4 administration at indicated concentrations for 24 h. Cell viability was calculated by CCK-8 assay. (b) QC4 activated oncosis marker calpain-1 autolysis from the 80 kDa event to the 76 kDa event. (c) QC4 degraded *β*-actin and *α*-tubulin. (d) Ultramicrostructure of SGC-7901 cells after 10 *μ*g/mL QC4 administration for indicated time. (e) QC4 activated the cleavage of apoptotic proteins. Carboplatin (CBP) was set as positive control (^*∗*^ *p* < 0.05 for the combination of QC4 with PD150506 treatment group against the QC4 alone treatment negative control group in SGC-7901; ^&^ *p* < 0.05 for the combination of QC4 with Z-VAD-FMK treatment group against the QC4 alone treatment negative control group in SGC-7901; ^#^ *p* < 0.01 for the combination of QC4 with PD150506 treatment group against the QC4 alone treatment negative control group in MGC80-3; ^$^ *p* < 0.05 for the combination of QC4 with Z-VAD-FMK treatment group against the QC4 alone treatment negative control group in MGC80-3).

After confirming the cytotoxic effect of QC4 on gastric cancer cells, we sought to undercover the detailed type of cell death caused by QC4. In this effort, necrosis inhibitor Necrostatin-1, apoptosis inhibitor Z-VAD-FMK, and oncosis inhibitor PD150606 were utilized in our further research. We found that Z-VAD-FMK and PD150606 could partially reverse cell death caused by QC4 while Necrostatin-1 had no apparent effect on cell death caused by QC4 (Figure 4(a)). Based on these results we speculated that QC4 might induce cell death through oncosis and apoptosis. Because calpains were reported to function in the process of oncotic cell death [14], we further detected calpain-1 in QC4-treated gastric cancer cells by Western Blotting. As had been reported by Suzuki and Sorimachi that the activation of calpain at the membrane included the dissociation of calpain subunits and two successive autolytic events (80 kDa and 76 kDa) [15], we found that calpain-1 autolyzed from the 80 kDa event to the 76 kDa event when treated with QC4, which might imply the activation of this protein during the oncotic cell death (Figure 4(b)). Researchers have found that cytoskeleton was one of the substrates of calpains and *β*-actin and *α*-tubulin was identified as calpain substrate during oncosis [14, 16]. We evaluated the protein levels of *β*-actin and *α*-tubulin in QC4-treated gastric cancer cells. Beta-actin and *α*-tubulin suffered a significant decrease in both cell lines dose-dependently (Figure 4(c)). The ultramicrostructural changes of SGC-7901 cells after 10 *μ*g/mL QC4 administration were captured by transmission electron microscopy. It revealed that after QC4 administration plasma membrane blebbing, endoplasmic reticulum dilation and mitochondrial swelling happened (Figure 4(d)). The above ultrastructure changes confirmed our hypothesis that QC4 induced oncosis in gastric cancer cells. In addition, because the apoptosis inhibitor Z-VAD-FMK could partially reverse cell death, apoptosis was also evaluated. Caspase-3, caspase-9, and PARP play crucial roles in apoptosis pathways. As illustrated in Figure 4(e), the apoptosis in gastric cancer cells was induced by QC4 which proved by the increase of cleaved form of caspase-3, caspase-9, and PARP during the protein detection.

### 3.4. QC4 Induced ATP Depletion, Mitochondrial Membrane Potential Decrease, ROS Generation, and Cytosolic Free Ca^2+^ Elevation in Gastric Cancer Cells

**Figure 5:**
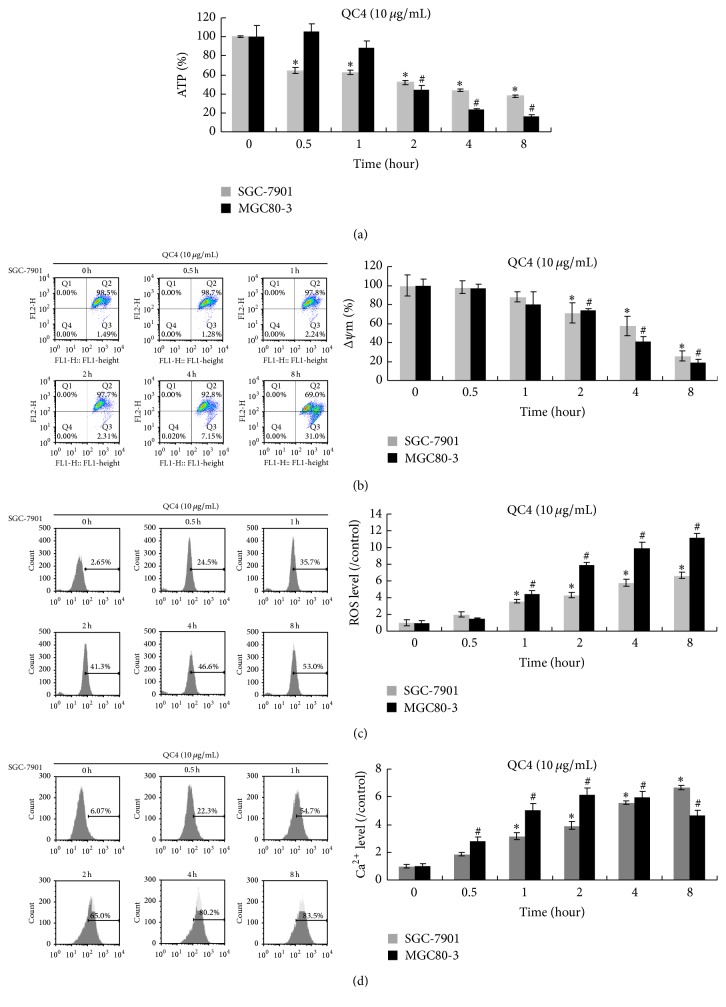
ATP depletion, mitochondrial membrane potential decrease, ROS generation, and cytosolic free Ca^2+^ elevation were observed in oncotic gastric cancer cells. ATP (a) and Δ*ψ*m (b) level generally decreased in 10 *μ*g/mL QC4-treated SGC-7901 and MGC80-3 cells time-dependently. ROS accumulation (c) and cytosolic free Ca^2+^ concentrations (d) were detected after 10 *μ*g/mL QC4 administration for indicated time in SGC-7901 and MGC80-3 cells (^*∗*^ *p* < 0.05 for QC4 treatment compared with the DMSO treated control group in SGC-7901; ^#^ *p* < 0.05 for QC4 treatment compared with the DMSO treated control group in MGC80-3).

Liu et al. have defined oncotic cell death as the point in the oncotic process in which respiration (mitochondrial function and associated ATP formation) and ion homeostasis could not be restored [14]. We further investigated ATP formation, mitochondrial function, and cytosolic free Ca^2+^ concentration in QC4-treated cells. ATP depletion was discovered in both cell lines after QC4 administration, and it was more obvious in MGC80-3 cells. After 8 h of administration, the ATP depletion in MGC80-3 cells was more than 80% (Figure 5(a)). As indicators of mitochondria damage, mitochondrial membrane potential and ROS were also detected. We found that mitochondrial membrane potential decreased time-dependently (Figure 5(b)) and the QC4-treated cells showed a tendency of ROS accumulation (Figure 5(c)). Moreover, the increasement of free Ca^2+^ was a key event in oncotic cell death. In our study, cytosolic free Ca^2+^ concentrations were elevated in both cell lines time-dependently (Figure 5(d)).

## 4. Discussion

Gastric cancer is a highly prevalent malignancy and the third material cause of cancer related death worldwide. Gastric cancer incidence has decreased substantially in many regions in the word due to the reductions in chronic* H. pylori* infection, increase in the availability of fresh food, an active screening program in district like Japan, and so forth. However, because of the characteristics of late emerged symptoms and thus often delayed interventions, gastric cancer still accounts for a considerable amount of mortality around the word, with the 5-year survival being less than 10% [2, 3]. For patients with late stage gastric cancer and those with local relapse or with distant metastases, additional chemotherapy and chemoradiotherapy have been proved to have survival benefits compared to surgery alone [17, 18]. Cisplatin and fluoropyrimidine-based chemotherapy are a widely used treatment for stage IV gastric cancer patients. As for patients with positive human epidermal growth factor receptor 2 (HER2) expressions, trastuzumab in combination with chemotherapy is considered as a new standard option [19]. Because the survival benefit for postoperative chemotherapy, chemoradiotherapy, and perioperative chemotherapy in case of pathological *T* > 2 and/or lymph node positive gastric cancer patients have been established, we believe that the development of novel anticancer agents would shed light on the prognosis of gastric cancer patients.

DHA is a natural occurring diterpene resin acid and is also a perfect template for structural modifications. Researches focusing on the synthesis and evaluation of new DHA derivatives are emerging in recent years. Many derivatives of DHA have been reported to have the property of inhibiting cancer cells and bacteria [9–12]. We demonstrated in the present work that the DHA derivative QC4 exerted strong cytotoxicity on gastric cancer cells. It totally inhibited cell viability at the concentration of 10 *μ*g/mL or higher after 48 h of administration. Furthermore, we focused on the specific cell death type caused by QC4 in gastric cancer cells. For the induced cell death could be partially reversed by oncosis inhibitor PD150606 and apoptosis inhibitor Z-VAD-FMK, QC4 induced cell death mainly by oncosis and apoptosis pathway. Meanwhile, oncosis was dominant in the process of QC4 induced cell death which proved by ultramicrostructure analysis.

Oncosis, which is originated from the word “swell,” is one type of accidental cell death. It is characterized by a series of early morphological changes and the ultimate cell membrane destruction and cell death. The early morphological changes include plasma membrane blebbing, endoplasmic reticulum dilation, mitochondrial swelling, and nuclear chromatin clumping [14]. Recently, oncosis regained researchers' attention as some agents exhibited anticancer activity via this process. In our research, we discovered that plasma membrane blebbing, endoplasmic reticulum dilation, and mitochondrial swelling happened in OC4-treated gastric cancer cells by transmission electron microscope. Calpain is a family of Ca^2+^-activated cysteine proteases that have a role in cell death. It has been reported that calpains implicate in the mediation of oncosis and the underlying mechanism of oncosis might be related to activation of calpain accompanied with the autolysis [20–22]. We studied the protein level of calpain-1 and found the autolysis of calpain-1 from the molecular weight of 80 kDa to 76 kDa which suggested the activation of calpain as well as the oncotic events.

In our study, we also detected decreased ATP level and impaired mitochondrial function in QC4-treated gastric cancer cells. The ATP depletion was thought to be the initial step towards oncosis. Liu et al. summarized that oncosis would occur when ATP depletion was greater than 80%–85% and the oncotic cell death could not be restored when the respiration and ion homeostasis were totally destroyed [14]. ATP depletion caused the inactivation of Na^+^-K^+^-ATP ase at the membrane which resulted in the disruption of ion homeostasis. The elevated free Ca^2+^ concentration could regulate a second mechanism that was responsible for mitochondrial membrane permeability. The impaired mitochondrial function further aggravated the situation of ATP depletion [23, 24]. In our study, the QC4-treated cells showed a significant elevated free Ca^2+^ concentration, while ROS accumulated and Δ*ψ*m collapsed as the marker of cellular damage. Finally, oncotic cell death was irreversible when mitochondrial function and ion homeostasis were totally destroyed.

In this work, we found that QC4 induced not only oncosis but also apoptosis cell death. Compared to oncosis, apoptosis is a more tightly controlled and orchestrated process. Signaling pathways that lead to apoptosis often include the extrinsic receptor-mediated pathway, the intrinsic mitochondrial-mediated pathway, caspase-2-dependent pathway, and caspase-independent pathway [25]. Many researches revealed some common pathways shared by oncosis and apoptosis. Lemasters found elevation of both oncosis and apoptosis markers in cell death [26]. Nakagawa and Yuan revealed the crosstalk between calpains and caspase families [27]. They found that m-calpain was responsible for cleaving procaspase-12 to generate active caspase-12. Moreover, calpasin could also cleave the loop region of Bcl-xL and turn it into a proapoptotic molecule. Procaspase-3 was one of the proteins that could be cleaved by both calpains and caspases [25]. In our research, we speculated that the activation of apoptosis might be attributed to the direct cleavage of caspases by calpains, because the activation of calpain-1 happened instantly after QC4 administration at earlier than 4 h and the activation of caspases followed the activation of calpain-1.

In conclusion, we found that the DHA derivative QC4 was capable of inhibiting gastric cancer cell viability by activating oncosis and apoptosis which would make QC4 a promising anticancer agent in the gastric cancer treatment.

## Figures and Tables

**Table 1:** The IC_50_ values of QC4 on SGC-7901 and MGC80-3 cells (*μ*g/mL).

	SGC-7901	MGC80-3
12 h	8.284885	8.170444
24 h	5.962502	5.403097
48 h	4.562062	4.752367

IC_50_: concentration at which cells were inhibited by 50%.
